# The development strategies of amateur table tennis matches in China based on the SWOT-AHP model: a case study in Shanghai

**DOI:** 10.1038/s41598-024-62334-2

**Published:** 2024-05-27

**Authors:** Qing Yi, Zuhong Liu, Xutao Liu, Yidan Wang, Rongzhi Li

**Affiliations:** 1 Faculty of Sports and Exercise Science, Universiti Malaya, Kuala Lumpur, Malaysia; 2 Department of Physical Education, Shanghai Sanda University, Shanghai, China; 3 Department of Studies, Faculty of Educational Studies, Universiti Putra Malaysia, Serdang, Malaysia; 4 China Table Tennis College, Shanghai University of Sport, Shanghai, China

**Keywords:** Amateur sports matches, Mass sports, Amateur matches development, Sports management, Public health, Leisure sport, Environmental social sciences, Signs and symptoms

## Abstract

Given the significance of amateur sports matches in health promotion and city culture construction. It is essential to systematically analyze the organizational mode of city amateur matches and propose development strategies. This study aimed to investigate the sustainable development strategies for city amateur matches in China. This study adopted a hybrid model of combined SWOT and the AHP analysis, using the Shanghai City Amateur Table Tennis Matches (ATTM) as a case study. Results showed that 20 factors of the SWOT analysis were included, and the ranking of weights of the SWOT group are Strengths, Opportunities, Weaknesses and Threats, respectively, and the strategic vector (θ, ρ) are (74.21°, 0.5861). ATTM should adopt the S–O pioneering strategy and leverages its advantages and opportunities to promote further development. The findings indicate that ATTM with advanced organizational mode, has good internal strengths and external opportunities, which can enlighten the development of amateur table tennis matches for other regions and countries. Future research should apply the hybrid model to a broader range of events and conduct comparative analyses across countries and regions.

## Introduction

Amateur sports matches are a vital component of sports competitions, serving as a fundamental avenue for participating in physical activity. They play a crucial role in increasing mass sports participation, improving physical fitness levels, and enhancing the well-being of participants^1^. In addition, conducting amateur matches could contribute to the promotion of mass sports culture^2,3^, and generate positive economic impacts for governments and the sports industry^4,5^. However, the current impacts of amateur sports matches did not meet the requirements for national fitness and sports power in China^6^. Therefore, there is an urgent imperative to promote further development of amateur sports matches in China.

Current research on amateur sports matches predominantly focuses on sports injury and rehabilitation^7,8^, amateur sports economy^9^, physiology and biochemistry^10,11^, and the promotion of participation^12,13^. Amateur table tennis matches are an essential component of amateur sports matches. They are affordable, can be played both indoors and outdoors, and have low age and fitness requirements, allowing accessible to all age groups. Existing studies primarily examine the social benefits of amateur table tennis matches^13^ and the sports biomechanics and physiology between amateur table tennis players and elite athletes^14^. However, there are very limited studies targeting the development strategies of amateur table tennis matches.

Table tennis was introduced to China in 1904, and Shanghai is the first city where table tennis entered. Table tennis has experienced rapid development in Shanghai since its introduction. Table tennis facilities are widespread throughout the city, and enterprises, schools and government agencies have established table tennis teams at various levels. By 1955, there were 7264 table tennis teams, 8701 tables, and 1745 competitions^15^. For elite table tennis, Shanghai is considered as the cradle of Chinese table tennis champions, has cultivated many outstanding athletes^16^. To date, Shanghai players have won at least 69 world championships. In addition, Shanghai has cultivated many renowned coaches. Recently, China Table Tennis College and International Table Tennis Federation Museum & China Table Tennis Museum have settled in Shanghai, which has brought new opportunities for the development of table tennis in Shanghai.

As the earliest city to introduce table tennis to China, and Shanghai takes the lead in organizing amateur table tennis matches^17^. ATTM is defined as a large mass table tennis league with the theme of “Play Together”, which can be divided into three levels. ATTM aims to address the contradiction between the people's growing demand for a better life and the unbalanced and insufficient development, to improve the city's amateur competition system, to meet the public's fitness needs, and to promote the development of table tennis in Shanghai. ATTM has an advanced tournament concepts and organizational and operational mode. Similarly, Japan has achieved significant success in the development both of amateur and elite table tennis. In Tokyo, for instance, there are many amateur table tennis matches such as the East League, the first and second half league season, which are also open to Tokyo residents, and also have an advanced organizational model^18^. However, amateur table tennis matches are poorly organised in most countries, which need to be improved. Undoubtedly, investigating the organizational and operational model of ATTM will give specific insights into other countries and cities in China.

In the field of management, SWOT and Analytic Hierarchy Process (AHP) analysis are useful tools for decision-making. AHP has been utilized in various fields and is increasingly applied in sustainability development, such as manufacturing^19^ and urban-related^20,21^ areas. Additionally, to improve the accuracy and applicability of decision-making, AHP has been combined with other decision-making tools and techniques, such as SWOT^22,23^, technique for the order of preference to the ideal solution (TOPSIS)^23^, preference ranking organisation method for enrichment evaluation (PROMETHEE)^24^, artificial neural networks (ANN)^25^, geographic information systems (GIS)^26^. Among these, the SWOT-AHP hybrid decision model^27,28^ is a common form, which has also been applied in the field of sports science^22,29–31^. Notable, this hybrid model has been applied to decision-making for major sports event and has yielded reliable results^31^. As a decision-making and management method, it is also suitable for analyzing the development of amateur table tennis matches. However, there is no study using this hybrid model on the development of amateur sports matches. Therefore, the SWOT-AHP hybrid model was adopted to examine the development of ATTM.

The present study employed the SWOT-AHP model to investigate the development of amateur sports matches with a case study of ATTM in China. The objectives of our study are to investigate ATTM’s advanced organization, planning, and implementation model and propose future development strategies. Therefore, this study not only identifies the current status of ATTM and outlines further development strategies, but also offers specific insights for other countries and regions in China.

## Methods

### Participants

Participants should be officially enrolled in the ATTM, and managers should be full-time or part-time race managers employed by the ATTM. Participants are required to understand the questionnaire and voluntarily participate in the investigation. Shanghai has 16 districts, consisting of seven urban districts, eight suburban districts, and one semi-urban semi-suburban district. For sampling purposes, this semi-urban and semi-suburban district is classified as an urban district. Therefore, we adopted stratified random sampling, with four urban districts and four suburban districts being randomly selected. Then, 2–4 matches were randomly selected in each district, and participants who met the inclusion criteria were selected for each match. Ultimately, 550 competitors and 80 managers were included from 30 matches.

### SWOT-AHP hybrid model

SWOT analysis is a strategic method employed to evaluate the strengths and weaknesses of a given situation, while also identifying both opportunities and threats by considering internal and external factors influencing the problem environment. This approach integrates positive and negative aspects from both internal and external sources to comprehensively assess the situation and promote success^32^. However, SWOT analysis merely provides a qualitative analysis, while few incorporate quantitative analyses. Additionally, due to the intricate nature of planning processes involving numerous criteria and interdependencies, this analytical method may not be adequately utilized^33,34^. Combining SWOT with AHP can overcome this issue.

AHP is a multi-criteria decision making (MCDM) technique developed by Saaty for analyzing complex decision-making problems. The process involves multiple stages: structuring the problem, pinpointing key decision-making factors, assessing the significance of these factors, and integrating the weights of all decision-making factors^35^. AHP offers several advantages, such as the versatility of its goal-setting options and its capacity for both qualitative and quantitative analysis of choice qualities^36^. Consequently, it structures the problem, identifies the decision-making factors, measures their importance, and synthesizes them all. Undoubtedly, AHP is one of the most practical MCDM methods^37^. In particular, when evaluated against other decision-making methods based on predetermined relevant criteria, AHP demonstrates significant effectiveness^38^. However, one of the main drawbacks of AHP is that it allows altering the generated “rankings”. In addition, several studies have reported the hierarchical structure lacks flexibility and is inadequate for modeling intricate scenarios^39,40^.

AHP can be combined with a SWOT analysis that lacks quantitative analysis^41,42^. One study demonstrates that SWOT and MCDA are commonly combined, where a mix of SWOT and AHP is most prevalent^41^. Each SWOT group can be formulated into a pairwise comparison matrix, and AHP is used to determine the weight of the SWOT factors^43^. The SWOT-AHP hybrid model also has been successfully applied in research in different areas^30,44^, including major sports events^31^.

#### Factors generation

**Figure 1:**
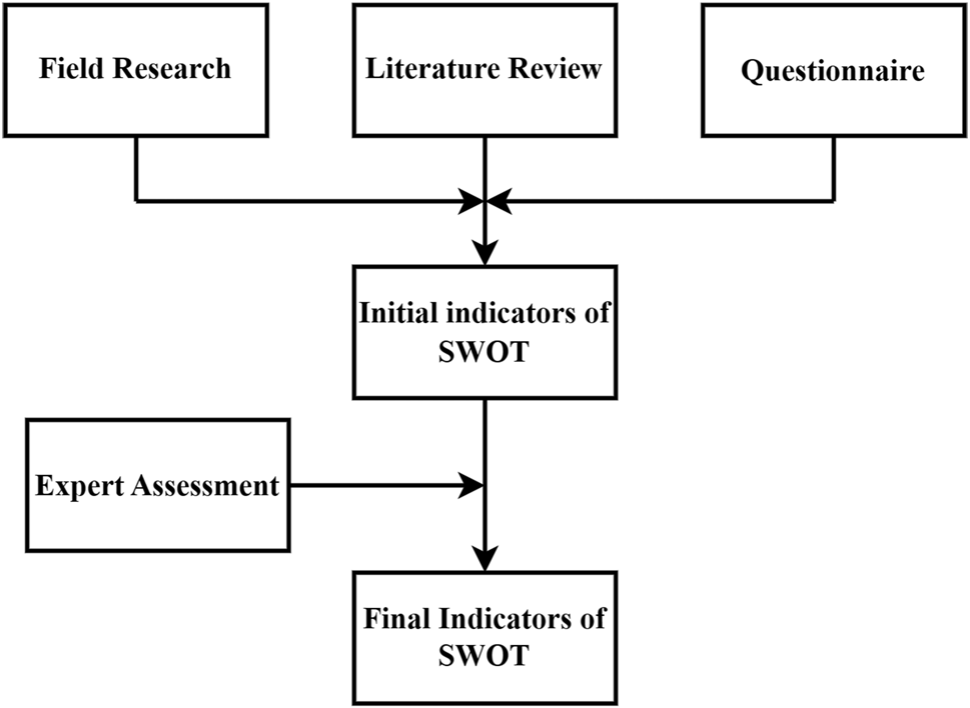
The SWOT indicator identifies the process.

Figure 1 illustrates the process of generating the SWOT factors. Specifically, we designed the “ATTM Development Status Questionnaire” which was distributed to participants and event managers to gather information about the participants' characteristics and their experiences. The questionnaire survey was conducted from July to October 2019. We also utilized search terms such as “amateur sports match*”, “mass sports match*”, and “mass sports event*” to search for relevant literature in WOS (Web of Science) and CNKI (China National Knowledge Infrastructure) to identify potential SWOT factors. Furthermore, five experts from the China Table Tennis College, the Shanghai University of Sport evaluated and screened the initially determined factors. All five are experts in the area of table tennis research, three are professors, two are associate professors, three hold PhD degrees, two hold master's degrees, and all possess over 10 years of experience in the field. Ultimately, we identified 20 factors that have the most direct impact on the development of the ATTM (Table 1).

**Table 1:** The factors of SWOT-AHP.

SWOT Group	SWOT Factor	Description of Factor
Strengths (S)	S1 The league system is more reasonable	The first stage is a team round-robin, and the second stage is a team elimination tournament
	S2 Low requirements for participation in the league	Available to all Shanghai residents (except for current professional club athletes and coaches) to meet the needs of different age groups
	S3 Regulatory agencies with oversight	The Shanghai City Amateur League Office supervises the league, and the five departments are the General Coordination Department, Competition and Activities Department, Publicity and Information Department, Marketing and Promotion Department, and Comprehensive Security Department
	S4 Long span of the league (January-December)	Matches are held almost every week throughout the year, guaranteeing participants' frequency of participation
	S5 Scientific and reasonable mode of organizing matches	Guided by the Shanghai Municipal Sports Bureau, the Table Tennis Association, and the Amateur League Office, events are jointly organized by district Table Tennis Associations, event managers, high-level referees, corporate employees, and others
Weaknesses	W1 The quality of C- League matches is uneven	Some of the C-Leagues are of high quality while others are of low quality
(W)	W2 Low level of service quality in the C-League	There are several issues, such as lack of tables, lack of seats in the rest area, no drinking water, and no signage to guide the participants
	W3 Low professional level of third-party evaluation agencies	Third-party evaluators lack sufficient expertise and understanding of the project, hindering in-depth evaluation
	W4 Insufficient investment in league funding	Funding is raised through the collaboration of the "government-society-market" triad, but it's insufficient
	W5 Insufficient promotion of the league	The league's visibility still needs to be improved; the publicity is not enough, and participants' awareness is not high
	O1 Strong support from the Shanghai (Municipal) Administration of Sports and Table Tennis Association	The Shanghai (Municipal) Administration of Sports organized the league, which plays a vital role in both pre-tournament bidding and post-tournament performance evaluation
	O2 Table tennis has a good mass base	The number of people involved in amateur table tennis in Shanghai is very high
Opportunities (O)	O3 Table tennis culture is more prosperous	The China Table Tennis College, International Table Tennis Federation Museum and China Table Tennis Museum located in Shanghai both play the role of talent training, cultural dissemination, and exhibition and scientific research
	O4 The continuous improvement of Shanghai venue facilities	The Shanghai Municipal Government has launched relevant policies to guarantee the construction and renewal of sports facilities
	05 Opportunity to build a globally renowned sports city	During the process of building a globally renowned sports city, government agencies place a high emphasis on reform and innovation for league
Threats (T)	T1 Diversion of different sports (homogeneous competition)	Badminton, swimming, basketball, and powerlifting have a diversionary effect
	T2 Lack of professional event managers	Fewer people are full-time sports event managers, while part-time managers make up the majority
	T3 Imbalance in the age distribution of participants	Most middle-aged and older adults participate in the league, while the proportion of young people involved is not optimistic
	T4 Fast-paced lifestyle in Shanghai	Shanghai's overloaded workload and paced lifestyle may limit their time for league participation
	T5Maintaining participant enthusiasm	The enthusiasm and stickiness of the participants to participate consistently are low and need to be improved

This study was waived for ethical approval according to the requirements of the ethical application guidelines of the Shanghai Sport University. All participants signed a written informed consent. In addition, this study was a low-risk sporting event investigation that did not involve sensitive information, and all subjects were ensured confidentiality and anonymity.

In addition, all methods were carried out following relevant guidelines and regulations.

#### SWOT-AHP model judgment matrix construction

**Figure 2:**
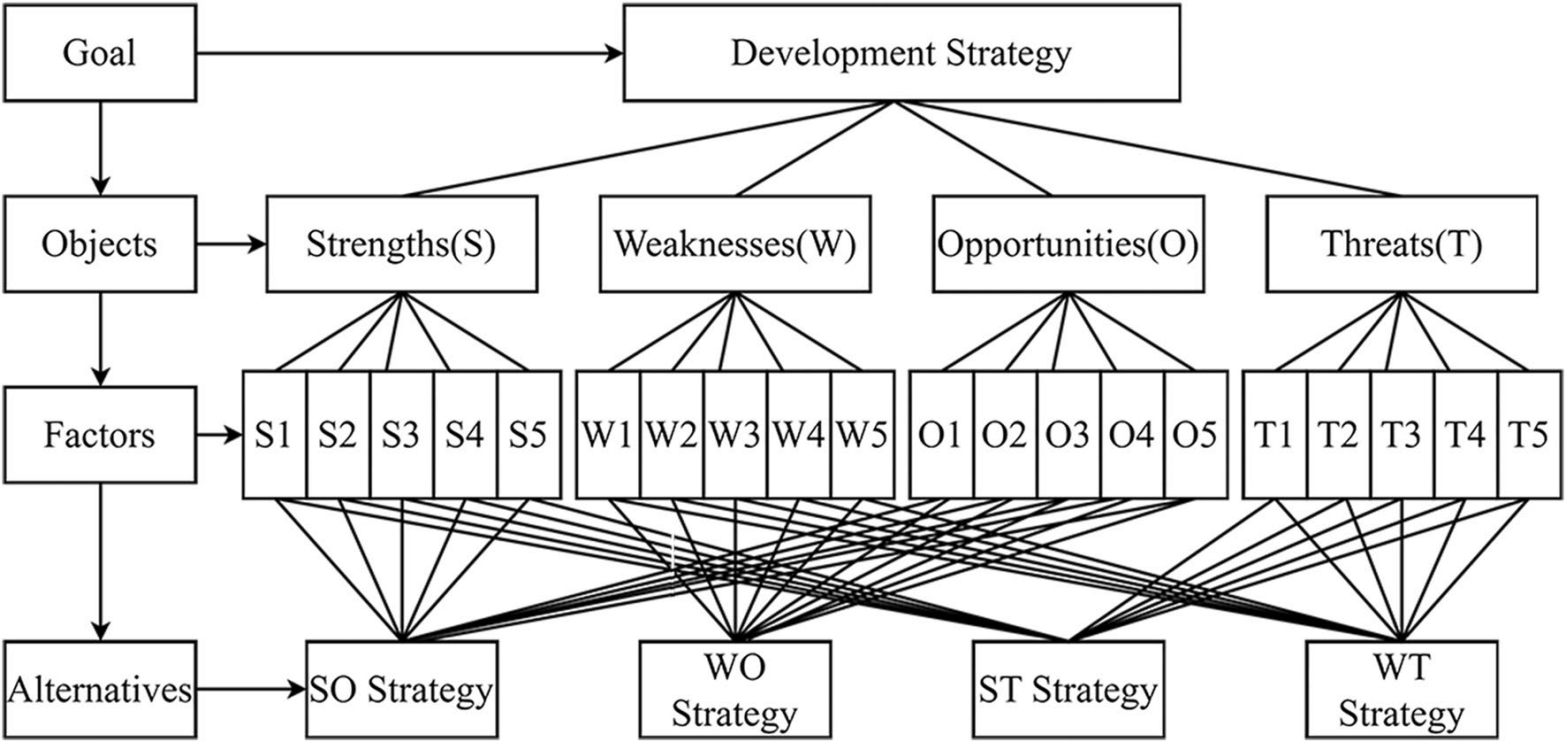
The hierarchical structure of SWOT-AHP.

Distributing the "ATTM Development Strategy Environment Expert Assignment Questionnaire" to the experts from China Table Tennis College, Shanghai University of Sport, using Saaty’s point scale^45^ (Table 2) to compare and assign values to each element in SWOT, that is, to complete the following discrimination: (1) Comparing the two factors (2) The specific assignment of the degree of importance of the two factors. The expert group employed Saaty’s point scale^45^ to compare two factors to each other to obtain the judgment matrix of S, W, O, and T. The constructed judgment matrix is as follows (Fig. 2).

**Table 2:** Scales for the pairwise comparisons.

Evaluation assignment	Uij importance level	Description
U = 1	Equally important	Ui and Uj are equally important
3	slightly important	Ui is slightly more important than Uj
5	Obviously important	Ui is significantly more important than Uj
7	Strongly Important	Ui is strongly more important than Uj
9	Extremely important	Ui is extremely more important than Uj
1/3	Slightly unimportant	Ui is slightly less important than Uj
1/5	Obviously unimportant	Ui is obviously less important than Uj
1/7	Strongly unimportant	Ui is strongly less important than Uj
1/9	extremely unimportant	Ui is extremely less important than Uj
2, 4, 6, 8 and its reciprocal		Take the middle value of the above two adjacent evaluation scales

#### Weight and consistency check

The consistency ratio (CR) is used to assess the effectiveness of measuring the responses of respondents, as they may give inconsistent answers. Yaahp software (Version 10.1) was used to carry out AHP and consistency tests and get the weight of each factor. The geometric mean was used to aggregate the individual priorities in group decision-making. The geometric mean was used due to it is considered to be more reliable in prioritization judgments^46^. Here are the steps and methods for calculating^22,45^:

Construct comparison matrix A:

The final ranking is presented in a weighted matrix, where each element aij (located in row i, column j) signifies the assigned relative weight. Correspondingly, its reciprocal (1/aij) is assigned as element aji. For any case where i = j, the value of aij  = 1.

$$\text{A}=\left({a}_{ij}\right)=\left[\begin{array}{cc}\begin{array}{c}\begin{array}{ccc}{a}_{11}& {a}_{12}& \cdots \end{array}\\ \begin{array}{ccc}{a}_{21}& {a}_{22}& \cdots \end{array}\\ \begin{array}{ccc}\vdots & \vdots & \ddots \end{array}\end{array}& \begin{array}{c}{a}_{1n}\\ {a}_{2n}\\ \vdots \end{array}\\ \begin{array}{ccc}{a}_{n1}& {a}_{n2}& \dots \end{array}& {a}_{nn}\end{array}\right]$$

Consistency test formula:

The maximum eigenvalue formulation is as follows:

$${\lambda }_{max}=\frac{1}{n}\sum_{i=1}^{n}\frac{AW}{{W}_{i}}$$

n represents the number of layers of the matrix, and Wi represents the value of the weight coefficient.

Combined with the formula to calculate the consistency index(CI) that can be used to analyze the deviation consistency index of each matrix:

$$CI=\frac{{\lambda }_{max}-n}{n-1}$$

CR: The calculation of CR requires a random index (RI), and the distribution of RI values is shown in Table 3:

**Table 3:** RI distribution table.

n	1	2	3	4	5	6	7	8	9	10	11	12	13	14
RI	0	0	0.58	0.90	1.12	1.24	1.32	1.41	1.45	1.49	1.52	1.54	1.56	1.48

The formula for calculating the consistency ratio is as follows:

$$CR=\frac{CI}{RI}$$

If CR ≤ 0.1, the consistency test is passed; on the contrary, if CR > 0.1, the consistency test is not passed.

#### Factor intensity and strategic quadrilateral

Factor intensity represents its magnitude of effect, and is determined by the estimated strength. Thus, the intensity can be calculated as the product of the estimated strength and weight: *intensity* = *estimated strength* × *weight*. The estimated strength is indicated on a scale of [− 4, 4], with positive values corresponding to S and O, and negative values corresponding to W and T. The higher the absolute value of the estimated strength, the greater the intensity. The aforementioned five experts were provided with the “Expert Questionnaire on the Intensity of Factors Influencing the Development of ATTM” to assess the intensity of each second layer factor. They assigned positive values to the factors that favored the development of ATTM and negative values to the factors that hindered the development of ATTM. The larger the absolute value of the positive value, the more favorable to the development of ATTM, while the negative value is the opposite.

The coordinate system is established based on the total strength of S, W, O, and T. The strategic quadrilateral can be created by connecting S′, W′, O′, and T′ on the respective half-axes of the coordinate system. S′, W′, O′, and T′ correspond to the total intensity of strengths, weakness, opportunities and threats, respectively.

#### The strategic vector (θ, ρ)

In the SWOT-AHP hierarchical structure model, the strategic azimuth (θ) can judge the strategic type, and the strategic strength coefficient can judge the strategy strength. In the polar coordinates of the strategic type and strategic strength spectrum, the coordinates (θ, ρ) form a strategic vector with θ as the azimuth and ρ as the polar radius.

The calculation procedures are as follows:

Coordinates of the center of gravity:

$${\text{P(X,Y)}} = {\text{P}}\left( {\sum \frac{{x_{i} }}{4},\sum \frac{{y_{i} }}{4}} \right)$$

Calculation of strategic azimuth:

$$\theta = {\text{arctan Y}}/{\text{X }}\left( {0 \le \theta \le \pi } \right)$$

where the xi and yi are the coordinates of S′, W′, O′, and T′ in the strategic quadrilateral, respectively.

The calculation of strategic positive intensity is:

$${\text{U}} = {\text{S}}{\prime} \times {\text{O}}{\prime}$$

The calculation of strategic negative intensity is:

$${\text{V}} = {\text{W}}{\prime} \times {\text{T}}{\prime}$$

The strategic intensity coefficient is:

$$\rho = {\text{U}}/\left( {{\text{U}} + {\text{V}}} \right)$$

The strategic strength coefficient (ρ) values range from 0 < ρ < 1, which can reflect the implementation strength of the strategy type, and it is generally judged in combination with 0.5. When the ρ > 0.5, pioneering strategies can be adopted, when the ρ < 0.5, a conservative strategy can be adopted.

## Results

### Weights of factors

Regarding the first layer factors, Table 4 shows that the internal strengths (0.5269) was the most influential factor, followed by external opportunities (0.2760), internal weaknesses (0.1174), and external threats (0.0798). Under strengths, the scientific and reasonable mode of organizing matches (S5) was the most influential factor, while the long span of the league (S4) ranked last. Under weaknesses, insufficient investment in league funding (W4) was the most important factor, while the low professional level of third-party evaluation agencies (W3) was the least important factor. Under opportunities, strong support from the Shanghai (Municipal) Administration of Sports and Table Tennis Association (O1) was the highest-rated factor, while, opportunity to build a globally renowned sports city (O5) was rated lowest. In the threats category, maintaining participant enthusiasm (T5) was the highest-rated factor, while diversion of different sports (homogeneous competition) (T1) had least impact.

**Table 4:** Weights and intensity of SWOT factors.

Weight of first layer factors	Weight of second layer factors	CR	Estimated strength	Factor intensity	Total intensity
Strengths (0.5269)	WS1 = 0.2725	0.0520	4	1.0900	∑Si = 3.5972
	WS2 = 0.0782		3	0.2346	
	WS3 = 0.1693		3	0.5079	
	WS4 = 0.0519		1	0.0519	
	WS5 = 0.4282		4	1.7128	
Weaknesses (0.1174)	WW1 = 0.0833	0.0431	− 3	− 0.2499	∑Wi = − 3.3550
	WW2 = 0.1336		− 3	− 0.4008	
	WW3 = 0.0485		− 1	− 0.0485	
	WW4 = 0.4520		− 4	− 1.8080	
	WW5 = 0.2826		− 3	− 0.8478	
Opportunities (0.2760)	WO1 = 0.4330	0.0314	4	1.7320	∑Oi = 3.5105
	WO2 = 0.2707		4	1.0828	
	WO3 = 0.0871		2	0.1742	
	WO4 = 0.1562		3	0.4686	
	WO5 = 0.0529		1	0.0529	
Threats (0.0798)	WT1 = 0.0613	0.0170	− 4	− 0.2452	∑Ti = − 2.6585
	WT2 = 0.2847		− 3	− 0.8541	
	WT3 = 0.1308		− 1	− 0.1308	
	WT4 = 0.1412		− 2	− 0.2824	
	WT5 = 0.3820		− 3	− 1.1460	

### Intensities of factors

As shown in Table 4, the total intensity analysis reveals that internal total strength intensity (3.5972) had the highest effect on the development of the ATTM, followed by external total opportunity intensity O (3.5105), internal total weakness intensity W (− 3.3550), and the external total threat intensity T (− 2.6585). The first and last ranked intensity of the strengths, weaknesses, and opportunities categories are ranked in the same order as their corresponding weights. In threats category, the maintaining participant enthusiasm (T5) was the highest-rated factor, while the imbalance in the age distribution of participants (T3) ranked last.

### SWOT strategic quadrilateral

**Figure 3:**
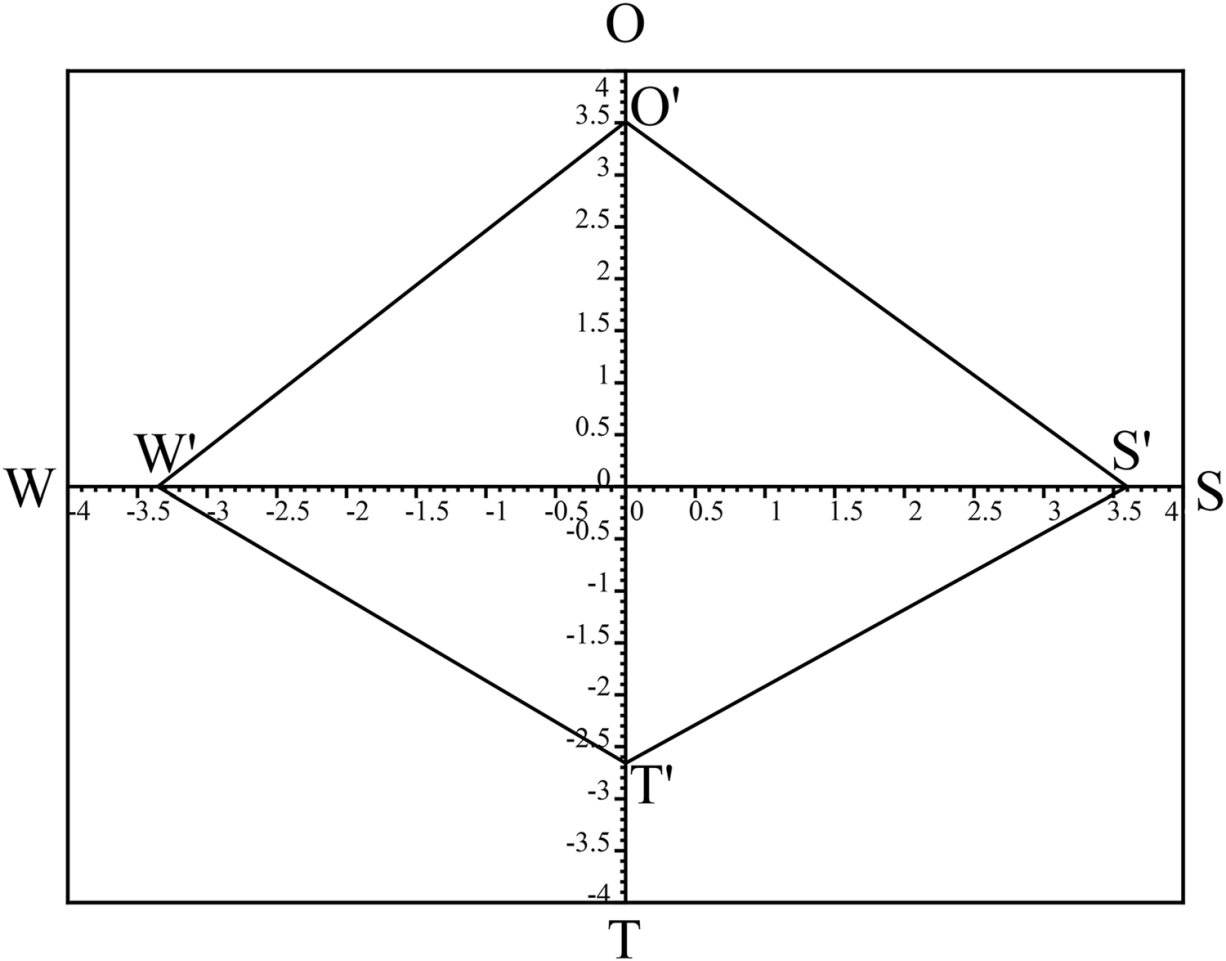
SWOT strategic quadrilateral.

Based on the total intensities of each group, the strategic quadrilateral was presented in Fig. 3. The values used for the calculations are from Table 4.

$$\sum {\text{ Si }} = { 3}.{5972}, \, \sum {\text{ Oi }} = { 3}.{51}0{5,}\quad \sum {\text{ Wi }} = \, - {3}.{355}0,\quad \sum {\text{ Ti }} = \, - {2}.{6585}$$

### Strategic vector (θ, ρ) and strategic type

The results indicated that the center of gravity coordinates (P) = (0.0606, 0.2130) ; the strategic azimuth (θ) = 74.21°; the strategic positive intensity (U) = 12.6280; the strategic negative intensity (V) = 8.9193; the strategic strength coefficient (ρ) = 0.5861, and the strategic vector (θ, ρ) = (74.21°, 0.5861).

**Figure 4:**
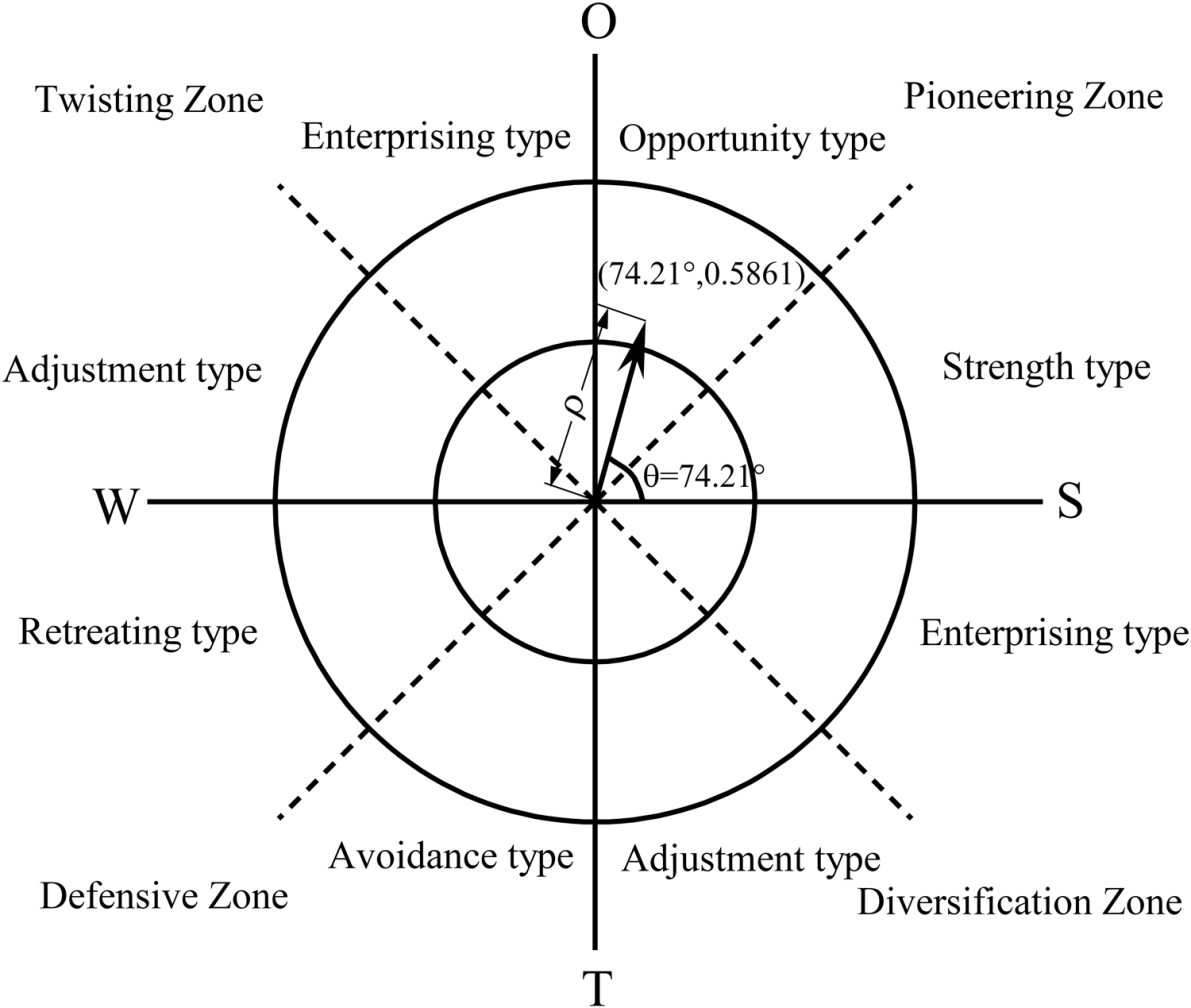
Strategic type and strategic intensity.

Since ρ > 0.5 and the strategic vector (θ, ρ) is (74.21°, 0.5861), the analysis combined with Fig. 4 indicates that the ATTM should adopt the S–O pioneering strategy, leveraging its advantages and opportunities. Specifically, ATTM should give full play to the advantages of the Shanghai (Municipal) Administration of Sports, the Table Tennis Association, and the resource allocation advantages of amateur league offices; continue to optimize the way of running the league and the tournament system; to improve the construction of venue facilities; to improve the number and age structures of participants by leveraging the opportunity of building a globally famous sports city; to construct the culture of table tennis in Shanghai by leveraging the positive role of the International Table Tennis Federation Museum and China Table Tennis Museum and the China Table Tennis College located in Shanghai.

## Discussion

This study found that the scientific and reasonable mode of organizing matches (S5) is the most influential factor in the strengths category. The results confirm the importance of the scientific model of event organization, in line with previous studies^47,48^. A study indicated that the organizational resources of sports events, such as strategic networks and partnerships, are key to guaranteeing their success^48^. Formulated under the guidance of the Shanghai (Municipal) Administration of Sports, the Table Tennis Association, and the Amateur League office, this method demonstrated a scientific approach to organizing matches. The second highest factor is that the league system is more reasonable (S1), which refers to the rules and specific arrangements of the match, such as the round-robin system and the home and away system^49^. The rationality of the match system has a vital role in the league, which will affect participant’s motivation and re-participation intention.

Under weakness category, insufficient investment in league funding (W4) was identified as the most influential issue, consistent with previous studies. Adequate funding is essential to enhance the quality and scale of the event^48^. For large-scale sports events, adequate financial support is not only the foundation of a successful event but also a guarantee of risk management^50^. The common issue of insufficient funding for amateur sports events limits their development and enhancement^51^. The possible reasons are related to the fact that governments usually invest a large amount of public sports funding into major sports or elite sports events^52^. ATTM adopted a "government-society-market" funding strategy, and the government funding model combines the purchase of public sports services with performance incentives, providing a maximum funding of 800,000 RMB^53^. However, ATTM with a long duration and a large number of participants, relying on government funding alone is insufficient. Given the widespread issue of inadequate funding, event organizers and local associations must explore diversified financial support strategies, among which commercial sponsorship is an effective strategy^54^.

The strong support from the Shanghai (Municipal) Administration of Sports and the Table Tennis Association (O1) is the highest impact factor in the opportunities category. Our findings are supported by a study that also reported the crucial contribution of the Shanghai Table Tennis Association in organizing and innovating table tennis matches^16^. In China, the robust leadership and coordination of the Chinese government authorities will ensure the success of the event to the greatest extent^55^. To ensure the quality and sustainable development of the Shanghai City Amateur League, the Shanghai (Municipal) Administration of Sports organized the ATTM, which plays a vital role in pre-tournament bidding and post-tournament performance evaluation^53^. Additionally, the Shanghai Table Tennis Association hosted the ATTM and also played a vital role in assigning referees and managers and coordinating the competition venues.

The highest-rated factor in the threats category was maintaining participant enthusiasm (T5). To attract residents to continue to participate, it is necessary to maintain the enthusiasm of already participating residents^56^. The key to maintaining the participants’ enthusiasm is to increase their satisfaction and motivation. Research has proven that human resources are important for the management and success of sports events^50^, which is consistent with our study. Our study identifies the lack of professional event managers (T2) as the second-ranked threat. Although the impact of professional management on sports event success is well-documented, research predominantly focuses on major events, with amateur events receiving less attention. As with large-scale sports events, sufficient professional event managers could similarly improve professionalism and service quality in amateur events.

This study indicated that ATTM should adopt a pioneering strategy that maximizes existing development advantages and opportunities. Adopting a pioneering strategy can improve the service quality of ATTM, and studies have reported that service quality is one of the most important predictors of satisfaction and retention^57^. The finding is consistent with the current emphasis on amateur sports matches by the Chinese government, ongoing economic and social development trends, and the evolving improvement of policies and regulations concerning amateur sports events. In addition, to ensure the further development of ATTM, internal disadvantages and external threats that hinder its development should be overcome and circumvented. Therefore, other countries and regions should fully leverage local strengths and opportunities in developing their amateur sports events.

### Limitations and future research

Firstly, due to the impact of the COVID-19 pandemic, this study only included data from the ATTM in 2019. It was not possible to compare data from different years, which reduced the timeliness of the study. Secondly, this study involved limited number of experts to a SWOT factors, which may limit the findings' generalizability. Therefore, the number of invited experts should be expanded to make the results more credible. Lastly, the novelty of amateur sports matches as a research topic in China means there are insufficient domestic academic studies available.

In the future, the range of experts should be expanded to include stakeholders such as sponsors and policymakers to provide multiple perspectives for strategic decision-making. Secondly, the SWOT-AHP hybrid model should be applied to various sports events, and SWOT analysis should be combined with different MCDA methods for comparative analysis. Thirdly, comparative studies of amateur sports events across regions and countries should be conducted to understand the influence of cultural, economic, and policy differences on event development. Lastly, research on motivations, satisfaction, and re-participation intentions for amateur sports events should be conducted to inspire organizers.

## Conclusions

Present study adopted the SWOT-AHP hybrid model to examine the development of amateur table tennis matches in China, using the ATTM as a case study. The results demonstrated that the SWOT model integrates 20 factors, and the strengths and opportunities have more influence than the weaknesses and threats (Supplementary file). ATTM should adopt the S–O pioneering strategy that combined the strengths with the opportunities. The findings can provide implications for the development of ATTM, and amateur table tennis matches in other regions and countries. Future research should incorporate more experts in diverse areas, and the SWOT-AHP model should be applied to more sports events management. Additionally, more studies should explore the development of amateur sports matches across different countries and regions, and how to improve participation motivation and satisfaction.

### Supplementary Information

Supplementary Information.

## Data Availability

All data are included in the manuscript along with supporting information.

## Reprints and permissions information

Is available at www.nature.com/reprints.
