# Supplementary material for: The development strategies of amateur table tennis matches in China based on the SWOT-AHP model: a case study in Shanghai
Source: Sci Rep. 2024 May 27;14:12060. doi: 10.1038/s41598-024-62334-2 (PMC11130325; doi:10.1038/s41598-024-62334-2)
Supplement: Supplementary file 1 — Supplementary Information. [file 41598_2024_62334_MOESM1_ESM.pdf]

ATTM development strategy

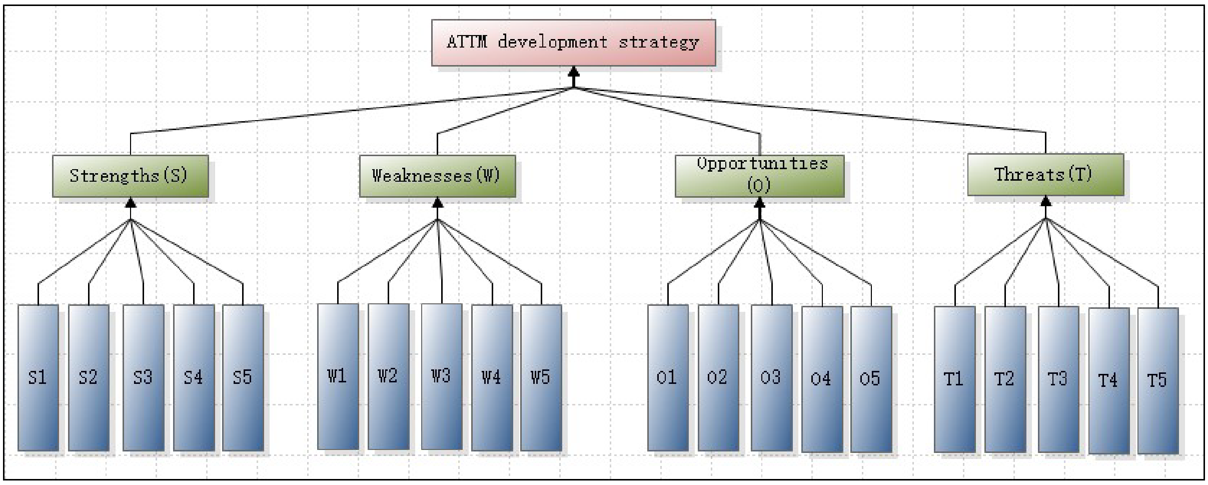

Scale Type:1-9.

Group decision-making - expert data aggregation method: weighted geometric mean of each expert's judgment matrix.

The ranking Weight of the elements in the objects layer to the decision goal

Objects	Weight
Strengths(S)	0.5269
Opportunities(O)	0.2760
Weaknesses(W)	0.1174
Threats(T)	0.0798

0. 1. Aggregated judgment matrices---ATTM development strategy Consistency Ratio (CR): 0.0403; Weight on "ATTM development strategy"： 1.0000

ATTM development strategy	Strengths(S)	Weaknesses(W)	Opportunities(O)	Threats(T)	Wi
Strengths(S)	1.0000	4.7894	2.7019	4.7429	0.5269
Weaknesses(W)	0.2088	1.0000	0.3147	2.0000	0.1174
Opportunities(O)	0.3701	3.1777	1.0000	3.7764	0.2760
Threats(T)	0.2108	0.5000	0.2648	1.0000	0.0798

0.2. Aggregated judgment matrices---Strengths(S) Consistency Ratio (CR): 0.0520; Weight on" ATTM development strategy"： 0.5269

Strengths(S)	S1	S2	S3	S4	S5	Wi
S1	1.0000	4.3174	2.7663	4.6821	0.3615	0.2725
S2	0.2316	1.0000	0.3413	2.0000	0.2187	0.0782
S3	0.3615	2.9302	1.0000	4.3379	0.3413	0.1693
S4	0.2136	0.5000	0.2305	1.0000	0.1818	0.0519
S5	2.7663	4.5731	2.9302	5.5016	1.0000	0.4282

0.5. Aggregated judgment matrices---Weaknesses(W)Consistency Ratio (CR): 0.0431; Weight on" ATTM development strategy"： 0.1174

Weaknesses (W)	W1	W2	W3	W4	W5	Wi
W1	1.0000	0.5296	2.3522	0.1997	0.2205	0.0833
W2	1.8882	1.0000	3.5652	0.2872	0.3010	0.1336
W3	0.4251	0.2805	1.0000	0.1556	0.2013	0.0485
W4	5.0080	3.4822	6.4282	1.0000	2.7663	0.4520
W5	4.5359	3.3227	4.9673	0.3615	1.0000	0.2826

0.6. Aggregated judgment matrices---Opportunities(O) Consistency Ratio (CR): 0.0314; Weight on" ATTM development strategy": 0.2760

Opportunities (O)	O1	O2	O3	O4	O5	Wi
O1	1.0000	2.3522	4.5359	3.1037	5.9663	0.4330
O2	0.4251	1.0000	3.3659	2.7019	4.7429	0.2707
O3	0.2205	0.2971	1.0000	0.3920	2.1689	0.0871
O4	0.3222	0.3701	2.5508	1.0000	3.3659	0.1562
O5	0.1676	0.2108	0.4611	0.2971	1.0000	0.0529

0.7. Aggregated judgment matrices---Threats(T) Consistency Ratio (CR): 0.0170; Weight on "ATTM development strategy": 0.0798

Threats(T)	T1	T2	T3	T4	T5	Wi
T1	1.0000	0.2648	0.3615	0.3494	0.1986	0.0613
T2	3.7764	1.0000	3.0639	2.2151	0.6084	0.2847
T3	2.7663	0.3264	1.0000	1.0000	0.3299	0.1308
T4	2.8619	0.4514	1.0000	1.0000	0.3594	0.1412
T5	5.0363	1.6438	3.0314	2.7821	1.0000	0.3820

1.1 Expert ID：1; Expert weight：0.2000---ATTM development strategy Consistency Ratio (CR): 0.0732; Weight：1.0000; λmax: 4.1954

ATTM development strategy	Strengths(S)	Weaknesses(W)	Opportunities(O)	Threats(T)	Wi
Strengths(S)	1.0000	7.0000	3.0000	4.0000	0.5515
Weaknesses(W)	0.1429	1.0000	0.3333	2.0000	0.1061
Opportunities(O)	0.3333	3.0000	1.0000	4.0000	0.2589
Threats(T)	0.2500	0.5000	0.2500	1.0000	0.0834

1.2 Expert ID：1; Expert weight：0.2000---Strengths(S) Consistency Ratio (CR):0.0380;Weight：0.5515; λmax: 5.1703

Strengths(S)	S1	S2	S3	S4	S5	Wi
S1	1.0000	3.0000	2.0000	3.0000	0.3333	0.2346
S2	0.3333	1.0000	0.5000	2.0000	0.2500	0.0979
S3	0.5000	2.0000	1.0000	4.0000	0.5000	0.1894
S4	0.3333	0.5000	0.2500	1.0000	0.2000	0.0620
S5	3.0000	4.0000	2.0000	5.0000	1.0000	0.4161

1.3 Expert ID：1; Expert weight：0.2000---Weaknesses(W) Consistency Ratio (CR): 0.0466; Weight：0.1061; λmax: 5.2088

Weaknesses (W)	W1	W2	W3	W4	W5	Wi
W1	1.0000	0.5000	3.0000	0.2000	0.2500	0.0875
W2	2.0000	1.0000	4.0000	0.2500	0.3333	0.1334
W3	0.3333	0.2500	1.0000	0.1429	0.1667	0.0425
W4	5.0000	4.0000	7.0000	1.0000	3.0000	0.4693
W5	4.0000	3.0000	6.0000	0.3333	1.0000	0.2673

1.4 Expert ID：1; Expert weight：0.2000---Opportunities(O) Consistency Ratio (CR): 0.0833; Weight：0.2589; λmax: 5.3732

Opportunities(O)	O1	O2	O3	O4	O5	Wi
O1	1.0000	3.0000	4.0000	2.0000	5.0000	0.3945
O2	0.3333	1.0000	3.0000	4.0000	6.0000	0.2984
O3	0.2500	0.3333	1.0000	0.3333	2.0000	0.0855
O4	0.5000	0.2500	3.0000	1.0000	3.0000	0.1679
O5	0.2000	0.1667	0.5000	0.3333	1.0000	0.0537

1.5 Expert ID：1; Expert weight：0.2000---Threats(T) Consistency Ratio (CR): 0.0665;Weight：0.0834; λmax: 5.2978

Threats(T)	T1	T2	T3	T4	T5	Wi
T1	1.0000	0.2500	0.3333	0.1667	0.3333	0.0521
T2	4.0000	1.0000	5.0000	0.3333	3.0000	0.2785
T3	3.0000	0.2000	1.0000	0.2500	0.5000	0.0966
T4	6.0000	3.0000	4.0000	1.0000	3.0000	0.4335
T5	3.0000	0.3333	2.0000	0.3333	1.0000	0.1392

2.1 Expert ID：2; Expert weight：0.2000---ATTM development strategy Consistency Ratio (CR): 0.0182; Weight：1.0000; λmax: 4.0486

ATTM development strategy	Strengths(S)	Weaknesses(W)	Opportunities(O)	Threats(T)	Wi
Strengths(S)	1.0000	4.0000	2.0000	5.0000	0.4896
Weaknesses(W)	0.2500	1.0000	0.3333	2.0000	0.1264
Opportunities(O)	0.5000	3.0000	1.0000	4.0000	0.3054
Threats(T)	0.2000	0.5000	0.2500	1.0000	0.0786

2. 2 Expert ID：2; Expert weight：0.2000---Strengths(S) Consistency Ratio (CR): 0.0506;Weight：0.4896; λmax: 5.2265

Strengths(S)	S1	S2	S3	S4	S5	Wi
S1	1.0000	5.0000	3.0000	5.0000	0.3333	0.2761
S2	0.2000	1.0000	0.3333	2.0000	0.2000	0.0720
S3	0.3333	3.0000	1.0000	4.0000	0.3333	0.1571
S4	0.2000	0.5000	0.2500	1.0000	0.1429	0.0467
S5	3.0000	5.0000	3.0000	7.0000	1.0000	0.4481

2. 3 Expert ID：2; Expert weight：0.2000---Weaknesses(W) Consistency Ratio (CR): 0.0643; Weight：0.1264; λmax: 5.2882

Weaknesses (W)	W1	W2	W3	W4	W5	Wi
W1	1.0000	0.5000	2.0000	0.3333	0.2500	0.1051
W2	2.0000	1.0000	3.0000	0.5000	0.3333	0.1724
W3	0.5000	0.3333	1.0000	0.2500	0.5000	0.0798
W4	3.0000	2.0000	4.0000	1.0000	2.0000	0.3577
W5	4.0000	3.0000	2.0000	0.5000	1.0000	0.2850

2.4 Expert ID：2; Expert weight：0.2000---Opportunities(O) Consistency Ratio (CR): 0.0111; Weight：0.3054; λmax: 5.0497

Opportunities(O)	O1	O2	O3	O4	O5	Wi
O1	1.0000	2.0000	4.0000	3.0000	6.0000	0.4258
O2	0.5000	1.0000	3.0000	2.0000	4.0000	0.2591
O3	0.2500	0.3333	1.0000	0.5000	2.0000	0.0972
O4	0.3333	0.5000	2.0000	1.0000	3.0000	0.1590
O5	0.1667	0.2500	0.5000	0.3333	1.0000	0.0588

2.5 Expert ID：2; Expert weight：0.2000---Threats(T) Consistency Ratio (CR): 0.0381;Weight：0.0786; λmax: 5.1705

Threats(T)	T1	T2	T3	T4	T5	Wi
T1	1.0000	0.2500	0.3333	0.5000	0.2000	0.0610
T2	4.0000	1.0000	2.0000	4.0000	0.3333	0.2607
T3	3.0000	0.5000	1.0000	2.0000	0.5000	0.1729
T4	2.0000	0.2500	0.5000	1.0000	0.2500	0.0931
T5	5.0000	3.0000	2.0000	4.0000	1.0000	0.4124

3.1 Expert ID：3; Expert weight：0.2000---ATTM development strategy
Consistency Ratio (CR): 0.0297; Weight：1.0000; λmax: 4.0792

ATTM development strategy	Strengths(S)	Weaknesses(W)	Opportunities(O)	Threats(T)	Wi
Strengths(S)	1.0000	5.0000	3.0000	6.0000	0.5577
Weaknesses(W)	0.2000	1.0000	0.3333	2.0000	0.1124
Opportunities(O)	0.3333	3.0000	1.0000	4.0000	0.2594
Threats(T)	0.1667	0.5000	0.2500	1.0000	0.0705

3.2 Expert ID：3; Expert weight：0.2000---Strengths(S) Consistency Ratio (CR): 0.0883;Weight：0.5577; λmax: 5.3955

Strengths(S)	S1	S2	S3	S4	S5	Wi
S1	1.0000	5.0000	3.0000	6.0000	0.3333	0.2892
S2	0.2000	1.0000	0.2500	2.0000	0.2000	0.0705
S3	0.3333	4.0000	1.0000	4.0000	0.3333	0.1710
S4	0.1667	0.5000	0.2500	1.0000	0.2500	0.0556
S5	3.0000	5.0000	3.0000	4.0000	1.0000	0.4137

3.3 Expert ID：3; Expert weight：0.2000---Weaknesses(W) Consistency Ratio (CR): 0.0613;Weight：0.1124; λmax: 5.2745

Weaknesses (W)	W1	W2	W3	W4	W5	Wi
W1	1.0000	1.0000	2.0000	0.1429	0.1667	0.0717
W2	1.0000	1.0000	4.0000	0.2500	0.2000	0.1029
W3	0.5000	0.2500	1.0000	0.1250	0.1429	0.0398
W4	7.0000	4.0000	8.0000	1.0000	3.0000	0.4753
W5	6.0000	5.0000	7.0000	0.3333	1.0000	0.3103

3.4 Expert ID：3; Expert weight：0.2000---Opportunities(O) Consistency Ratio (CR): 0.0616; Weight：0.2594; λmax: 5.2759

Opportunities(O)	O1	O2	O3	O4	O5	Wi
O1	1.0000	3.0000	6.0000	4.0000	7.0000	0.4780
O2	0.3333	1.0000	4.0000	3.0000	5.0000	0.2527
O3	0.1667	0.2500	1.0000	0.3333	3.0000	0.0798
O4	0.2500	0.3333	3.0000	1.0000	4.0000	0.1455
O5	0.1429	0.2000	0.3333	0.2500	1.0000	0.0439

3.5 Expert ID：3; Expert weight：0.2000---Threats(T) Consistency Ratio (CR): 0.0435; Weight：0.0705; λmax: 5.1948

Threats(T)	T1	T2	T3	T4	T5	Wi
T1	1.0000	0.3333	0.5000	0.2500	0.1667	0.0577
T2	3.0000	1.0000	3.0000	2.0000	0.3333	0.2133
T3	2.0000	0.3333	1.0000	0.5000	0.2500	0.0940
T4	4.0000	0.5000	2.0000	1.0000	0.2000	0.1508
T5	6.0000	3.0000	4.0000	5.0000	1.0000	0.4842

4.1 Expert ID：4; Expert weight：0.2000---ATTM development strategy Consistency Ratio (CR): 0.0306; Weight：1.0000; λmax: 4.0818

ATTM development strategy	Strengths(S)	Weaknesses(W)	Opportunities(O)	Threats(T)	Wi
Strengths(S)	1.0000	3.0000	2.0000	4.0000	0.4564
Weaknesses(W)	0.3333	1.0000	0.3333	2.0000	0.1461
Opportunities(O)	0.5000	3.0000	1.0000	3.0000	0.3032
Threats(T)	0.2500	0.5000	0.3333	1.0000	0.0944

4. 2 Expert ID：4; Expert weight：0.2000---Strengths(S) Consistency Ratio (CR): 0.0389;Weight：0.4564; λmax: 5.1741

Strengths(S)	S1	S2	S3	S4	S5	Wi
S1	1.0000	4.0000	3.0000	5.0000	0.5000	0.2960
S2	0.2500	1.0000	0.3333	2.0000	0.2500	0.0825
S3	0.3333	3.0000	1.0000	4.0000	0.3333	0.1661
S4	0.2000	0.5000	0.2500	1.0000	0.1667	0.0511
S5	2.0000	4.0000	3.0000	6.0000	1.0000	0.4044

4. 3 Expert ID：4; Expert weight：0.2000---Weaknesses(W) Consistency Ratio (CR): 0.0313;Weight：0.1461; λmax: 5.1403

Weaknesses (W)	W1	W2	W3	W4	W5	Wi
W1	1.0000	0.5000	2.0000	0.2000	0.2500	0.0797
W2	2.0000	1.0000	3.0000	0.2500	0.3333	0.1262
W3	0.5000	0.3333	1.0000	0.1429	0.1667	0.0479
W4	5.0000	4.0000	7.0000	1.0000	3.0000	0.4744
W5	4.0000	3.0000	6.0000	0.3333	1.0000	0.2718

4.4 Expert ID：4; Expert weight：0.2000---Opportunities(O) Consistency Ratio (CR): 0.0355; Weight：0.3032; λmax: 5.1589

Opportunities(O)	O1	O2	O3	O4	O5	Wi
O1	1.0000	2.0000	5.0000	4.0000	6.0000	0.4372
O2	0.5000	1.0000	4.0000	3.0000	5.0000	0.2874
O3	0.2000	0.2500	1.0000	0.3333	2.0000	0.0782
O4	0.2500	0.3333	3.0000	1.0000	3.0000	0.1441
O5	0.1667	0.2000	0.5000	0.3333	1.0000	0.0532

4.5 Expert ID：4; Expert weight：0.2000---Threats(T) Consistency Ratio (CR): 0.0255; Weight：0.0944; λmax: 5.1144

Threats(T)	T1	T2	T3	T4	T5	Wi
T1	1.0000	0.2500	0.3333	0.5000	0.1667	0.0568
T2	4.0000	1.0000	3.0000	4.0000	0.5000	0.2813
T3	3.0000	0.3333	1.0000	2.0000	0.2500	0.1332
T4	2.0000	0.2500	0.5000	1.0000	0.2000	0.0843
T5	6.0000	2.0000	4.0000	5.0000	1.0000	0.4444

5.1 Expert ID：5; Expert weight：0.2000---ATTM development strategy
Consistency Ratio (CR): 0.0866; Weight：1.0000; λmax: 4.2313

ATTM development strategy	Strengths(S)	Weaknesses(W)	Opportunities(O)	Threats(T)	Wi
Strengths(S)	1.0000	6.0000	4.0000	5.0000	0.5711
Weaknesses(W)	0.1667	1.0000	0.2500	2.0000	0.1005
Opportunities(O)	0.2500	4.0000	1.0000	4.0000	0.2544
Threats(T)	0.2000	0.5000	0.2500	1.0000	0.0740

5.2 Expert ID：5; Expert weight：0.2000---Strengths(S) Consistency Ratio (CR): 0.0755;Weight：0.5711; λmax: 5.3383

Strengths(S)	S1	S2	S3	S4	S5	Wi
S1	1.0000	5.0000	3.0000	5.0000	0.3333	0.2660
S2	0.2000	1.0000	0.3333	2.0000	0.2000	0.0706
S3	0.3333	3.0000	1.0000	6.0000	0.2500	0.1646
S4	0.2000	0.5000	0.1667	1.0000	0.1667	0.0464
S5	3.0000	5.0000	4.0000	6.0000	1.0000	0.4524

5.3 Expert ID：5; Expert weight：0.2000---Weaknesses(W) Consistency Ratio (CR): 0.0630;Weight：0.1005; λmax: 5.2822

Weaknesses (W)	W1	W2	W3	W4	W5	Wi
W1	1.0000	0.3333	3.0000	0.1667	0.2000	0.0755
W2	3.0000	1.0000	4.0000	0.2500	0.3333	0.1411
W3	0.3333	0.2500	1.0000	0.1429	0.1667	0.0419
W4	6.0000	4.0000	7.0000	1.0000	3.0000	0.4715
W5	5.0000	3.0000	6.0000	0.3333	1.0000	0.2700

5.4 Expert ID：5; Expert weight：0.2000---Opportunities(O) Consistency Ratio (CR): 0.0161; Weight：0.2544; λmax: 5.0721

Opportunities(O)	O1	O2	O3	O4	O5	Wi
O1	1.0000	2.0000	4.0000	3.0000	6.0000	0.4225
O2	0.5000	1.0000	3.0000	2.0000	4.0000	0.2569
O3	0.2500	0.3333	1.0000	0.5000	2.0000	0.0959
O4	0.3333	0.5000	2.0000	1.0000	4.0000	0.1689
O5	0.1667	0.2500	0.5000	0.2500	1.0000	0.0558

5. 6 Expert ID：5; Expert weight：0.2000---Threats(T) Consistency Ratio (CR): 0.0300; Weight：0.0740; λmax: 5.1344

Threats(T)	T1	T2	T3	T4	T5	Wi
T1	1.0000	0.2500	0.3333	0.5000	0.1667	0.0564
T2	4.0000	1.0000	3.0000	5.0000	0.5000	0.2921
T3	3.0000	0.3333	1.0000	2.0000	0.2500	0.1310
T4	2.0000	0.2000	0.5000	1.0000	0.2000	0.0806
T5	6.0000	2.0000	4.0000	5.0000	1.0000	0.4399
